# Cellular aging beyond cellular senescence: Markers of senescence prior to cell cycle arrest *in vitro* and *in vivo*

**DOI:** 10.1111/acel.13338

**Published:** 2021-03-12

**Authors:** Mikolaj Ogrodnik

**Affiliations:** ^1^ Ludwig Boltzmann Research Group Senescence and Healing of Wounds Vienna Austria; ^2^ Ludwig Boltzmann Institute for Experimental and Clinical Traumatology in AUVA Research Center Vienna Austria; ^3^ Austrian Cluster for Tissue Regeneration Vienna Austria

**Keywords:** aging, cellular senescence, evolutionary biology, molecular biology of aging, molecular damage, theories of aging, wound healing

## Abstract

The field of research on cellular senescence experienced a rapid expansion from being primarily focused on *in vitro* aspects of aging to the vast territories of animal and clinical research. Cellular senescence is defined by a set of markers, many of which are present and accumulate in a gradual manner prior to senescence induction or are found outside of the context of cellular senescence. These markers are now used to measure the impact of cellular senescence on aging and disease as well as outcomes of anti‐senescence interventions, many of which are at the stage of clinical trials. It is thus of primary importance to discuss their specificity as well as their role in the establishment of senescence. Here, the presence and role of senescence markers are described in cells prior to cell cycle arrest, especially in the context of replicative aging and *in vivo* conditions. Specifically, this review article seeks to describe the process of “cellular aging”: the progression of internal changes occurring in primary cells leading to the induction of cellular senescence and culminating in cell death. Phenotypic changes associated with aging prior to senescence induction will be characterized, as well as their effect on the induction of cell senescence and the final fate of cells reviewed. Using published datasets on assessments of senescence markers *in vivo*, it will be described how disparities between quantifications can be explained by the concept of cellular aging. Finally, throughout the article the applicational value of broadening cellular senescence paradigm will be discussed.

## Graphical abstract

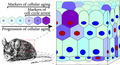

## 1 SYNONYMS OR DIFFERENT PROCESSES? CELLULAR AGING AND CELLULAR SENESCENCE INTRODUCED

The field of cellular senescence is currently one of the most rapidly developing branches of science. Its discoveries bear a great promise for effective treatments of age‐related diseases and human healthspan extension. However, as the field is still in its infancy, there is a lot of confusion regarding the characterization of cellular senescence and its position in organismal aging and physiology.

The beginning of the term “senescence” in the context of mammalian cell cultures is considered to have been born out of the discovery by Hayflick and Moorhead in 1961 (Hayflick & Moorhead, 1961). In their milestone article, the authors described that primary cells have a finite lifespan when cultured *in vitro*, contrasting cancer cells that divide without limits. Interestingly, the term “senescence”, as used in the original article, refers to an increase in cell degradation and accumulation of cell debris at the late stage of culture (termed “stage III” by the authors) (Hayflick & Moorhead, 1961). When it comes to the features of the “senescence stage,” the researchers described a reduction in mitotic activity and an increase in genomic instabilities. However, what caught the attention of the scientists was an increase in cell degeneration and debris, later described by the authors to resemble cell death (Hayflick, 1991). An observation of increased cell mortality bears certain similarity to a gradual increase in the risk of death of animals—a core feature of the aging process; therefore, a term “cellular senescence” was conceived. While it is possible that the regular splitting of late‐stage cells performed in this study contributed to the steep decline in cell number, other studies have confirmed a decrease in the number of senescent cells due to cell death (Fumagalli et al., 2014; Sitte et al., 2000; von Zglinicki et al., 1995) and cell death has been determined to be a primary consequence of senescence (Hayflick, 1991; Stanulis‐Praeger, 1987).

**FIGURE 1:**
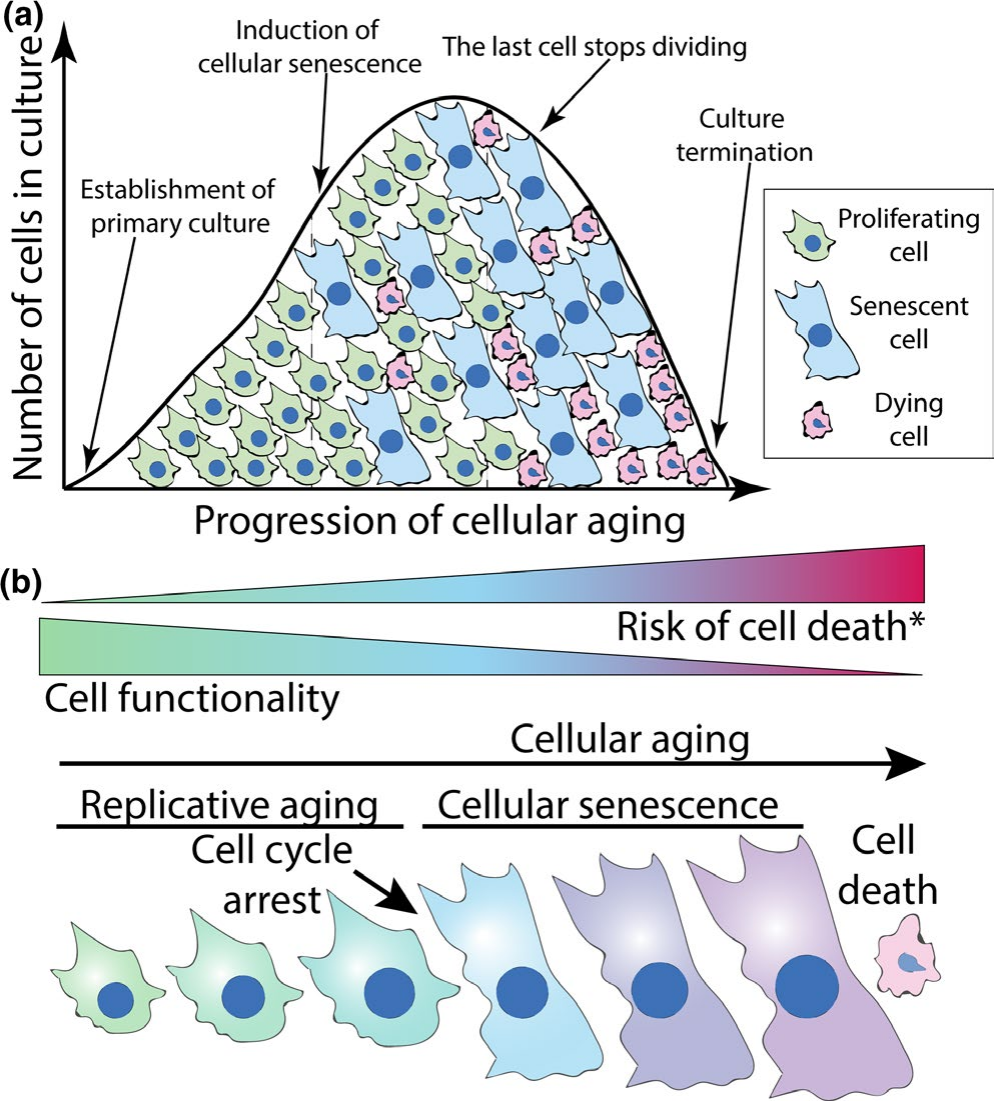
The concept of cellular aging. (A) The lifespan curve of primary cells in culture. After the culture establishment, cells enter into a phase characterized by exponential growth, which is followed by induction of senescence and a decline in growth rate. The final stage is a post‐senescence continuation of cellular aging and degradation of cell population. The graph simplifies cellular aging and does not show the transition gradient. (B) Cellular aging is a gradual process, which reduces cell functionality and increases risk of cell death over time. (*) The changes in the risk of death are unlikely to be linear, for example, cells shortly after senescence induction might be less prone to death than younger cells

Nowadays, the term cellular senescence is usually used to refer to an irreversible cell cycle arrest associated with changes in cell morphology, secretory profile, and epigenetic alterations among others (van Deursen, 2014; Gorgoulis et al., 2019). In the context of long‐term cultures of primary cells, where cells are continuously stimulated to proliferate (replicate), the maximum number of population doublings a culture of cells is able to attain is referred to as the “Hayflick limit,” the following irreversible cell cycle arrest is termed “replicative senescence” and the whole process is referred to as “replicative aging” (Liu et al., 2019). In contrast to the original findings (Hayflick & Moorhead, 1961), more recent research shows that cellular senescence is associated with a reduced sensitivity to cell death (Wang, 1995). How do these observations from more than half a century ago relate to the modern view on cellular senescence? One of the major differences is that the article by Hayflick and Moorhead describes the whole cellular “lifespan”: from the isolation of primary cells, through months of culture (6.48 months on average, up to a maximum of 11 months), with the “senescence stage” lasting several months and ending with the death of the cells (Hayflick & Moorhead, 1961). This contrasts the current research approach, where all the measurements and observations are done within a short period of time (days to weeks) after senescence induction. Therefore, it is likely that the landmark article (Hayflick & Moorhead, 1961) describes a much broader process of “cellular aging,” where “cellular senescence” is only one of the events preceding cell death (Figure 1a).

The term senescence in modern usage has come to require a permanent cell cycle arrest. In other words, even if other changes have occurred before it, a cell is not considered senescent until a permanent cell cycle arrest is attained. This assumption is not unjustifiable, as many major features of cellular senescence, such as the pro‐inflammatory phenotype, are dependent on the stable cell cycle arrest (Lopes‐Paciencia et al., 2019). However, since changes in primary cells occur in a gradual and continuous manner long before the cell cycle arrest, a new term is needed to encompass both these early changes as well as what happens after the onset of the permanent cell cycle arrest. Following the distinction between “replicative senescence” and “chronological aging” used in yeast (Longo et al., 2012), in this article the term “**cellular aging**” describes a gradual decline in cell function and an increase in probability of cell death, “**replicative aging**” refers to dividing cells prior to senescence induction, while “**cellular senescence**” is a cellular program initiated by the induction of a permanent cell cycle arrest that increases cell inflammation and prevents cell proliferation (Figure 1b). This article is focused on comparing cellular senescence with cellular aging and outlining the applicational value of their distinction in the context of *in vitro* and *in vivo* studies.

## 2 MARKERS OF CELLULAR SENESCENCE PRIOR TO SENESCENCE INDUCTION: EARLY EVENTS OF SENESCENCE INDUCTION OR EVIDENCE OF CELLULAR AGING?

The induction of cellular senescence is a binary program, meaning that there is a certain order of events occurring after a cell is induced to enter the stage of senescence, including the establishment of a permanent cell cycle arrest. “Binary” (or “bistable”) in this context means that for primary cells, the process is unidirectional and that there are no phenotypical stages preceding cell cycle arrest as cells cannot be half‐permanently arrested in cell cycle or be half‐senescent. Other binary programs include the initiation of the cell cycle, apoptosis, and oocyte maturation, among others as they operate on a basis of unidirectional commitment (Ferrell, 2013; Xiong & Ferrell, 2003; Yue & Lopez, 2020). An important feature of such binary programs is that there is a certain set of cellular machineries (and associated proteins, chromatin modifications, microRNAs etc.) dedicated for their execution, which can be used as “markers” of these processes. In practical terms, these markers can also be considered to be binary, that is, their expression is present or absent without significant in‐between phases. These include cyclins required for cell cycle progression or caspases required for apoptosis. Similarly to cell cycle progression and apoptosis, decades of research on cellular senescence has resulted in a set of features, “senescence markers” (van Deursen, 2014; Gorgoulis et al., 2019), associated with this binary program. However, in contrast to the previous examples, researchers agree that none of the markers is unique or specific to cellular senescence and that a combination of markers should be used to identify senescent cells (Gorgoulis et al., 2019). At the same time, the reason for why cellular senescence lacks specific and unique markers is often unclear.

One of the pieces of evidence for the alleged lack of specificity of senescence markers is their presence in primary cell cultures which have not yet reached the Hayflick limit. The most common explanation of this phenomenon is that some primary cells may undergo induction of cellular senescence earlier than others (i.e., prematurely), causing early cultures to contain a mixture of both young and senescent cells. Consistently, many studies have reported a heterogeneity of primary cultures (Absher & Absher, 1976; Nassrally et al., 2019; Passos et al., 2007; Smith & Whitney, 1980). For example, Passos et al., reported that up to 10% of cells in primary cultures are negative for proliferation markers and positive for markers of cellular senescence (Passos et al., 2007). However, more recent studies utilizing single‐cell transcriptomics found no evidence for a significant fraction of senescent cells among pre‐Hayflick's limit cultures, with senescent cells making up less than 1% (Tang et al., 2019; Wiley et al., 2017). Instead, a study by Tang *et al*. reported that not only are the transcriptomic profiles of cells approaching cellular senescence different from that of senescent cells, but they are also distinct from those of young cells (from cultures of low population doublings; PDs) (Tang et al., 2019). More specifically, the study utilized young (PD = 38), middle‐age (PD = 48), replicatively senescent (PD = 71) or stress‐induced prematurely senescent (SIPS) human fibroblasts and showed that young and middle‐age cells cluster in two distinct populations (Tang et al., 2019). Thus, the study provides a key piece of evidence on the existence of cellular aging. As single‐cell omics have only recently been used in cellular senescence research, more studies are needed to confirm these findings. However, it could be argued that these observations are due to a “bystander effect” (Nelson et al., 2012), defined as a detrimental effect of senescent cells on the phenotype of young cells (Box 1). Nevertheless, this does not contradict the criteria of cellular aging: non‐senescent, proliferating cells display gradual phenotypic changes prior to senescence induction, regardless of whether their origin is intracellular or external. Below, the markers of senescence in the context of cellular aging are characterized.

## 3 CELLULAR SENESCENCE AND CELLULAR AGING *IN VITRO*

### 3.1 Prolonged cell cycle

A connection between proliferation and cellular senescence has been explored in detail in cells undergoing senescence induction and establishment, while the proliferative properties of cells in pre‐Hayflick limit cultures have received much less research attention. If the only phenotypic change occurring in cultures of primary cells is a binary event occurring at the Hayflick limit, it could be expected that cells keep dividing unperturbedly until they reach that limit and enter senescence. This is, however, not the case and multiple studies have reported a gradual reduction in the proliferative capacity of cells in cultures prior to reaching the Hayflick limit (Absher et al., 1974; Kim, Byun, et al., 2013; Macieira‐Coelho & Azzarone, 1982; Nassrally et al., 2019; Ponten et al., 1983; Smith & Whitney, 1980). Specifically, early studies have shown that with an increasing passage number, cells gradually loose not only clonal capacity (number of progenies derived from a single clone) (Ponten et al., 1983; Smith & Whitney, 1980), but also increase the duration of the cell cycle (Absher et al., 1974; Nassrally et al., 2019) with a concomitant decline in the rate at which cells enter the S phase (Macieira‐Coelho & Azzarone, 1982). For example, Kim et al. reported that the population doubling time of human diploid fibroblasts (HDFs) starts increasing at around PD40 and keeps increasing until ~PD90 when cells enter cellular senescence (Kim, Byun, et al., 2013). One possible explanation for this phenomenon would be that some cells enter senescence stochastically and/or prematurely, resulting in an early‐onset sub‐population of senescent cells in a population of otherwise young cells. Methods commonly used in aging research rely on averaging the measurements from individual cells to express the results as a mean of the whole population. Such methodology would not be able to distinguish between a gradual decline in the proliferative capacity of all cells and a decline in proliferation in a population of proliferating cells mixed with a smaller fraction of non‐proliferating, senescent cells. This, however, can be distinguished using single‐cell approaches (Absher et al., 1974; Nassrally et al., 2019). Long‐term, live‐cell imaging (termed “time‐lapse cinematography” in older literature) revealed that there is an increase in division time in human primary fibroblasts prior to senescence induction (Absher et al., 1974; Nassrally et al., 2019). Specifically, Absher *et al*. reported that doubling time of virtually all recorded cells increases from 16.8 hours to 32.0 hours for WI38 fibroblasts between passage 28 and passage 53 (Absher et al., 1974). In addition, the study reported that there is an increase in the number of non‐dividing cells and a decrease in clonal capacity between early‐ and late‐passage cultures (Absher et al., 1974). Notably, the non‐dividing cells were not taken into consideration for assessment of doubling time; thus, the study showed that primary cells present features of aging prior to and independent from cellular senescence. Similar conclusions can be drawn from the recent study by Nassrally et al., where authors documented in two types of human primary fibroblasts that late‐passage cells continue cycling with extended cycle times (Nassrally et al., 2019). The authors also observed an increase in the fraction of non‐dividing cells and an increase in stochastic cell death in late passages. Based on these and other findings, the conceptual framework has been drawn describing aging of human fibroblasts as a succession of subtle changes in the cell cycle time and frequency (Macieira‐Coelho, 2010; Macieira‐Coelho & Azzarone, 1982; Macieira‐Coelho & Taboury, 1982). Overall, the experimental data from these studies suggest that in addition to a binary phenotype of cell cycle ablation associated with cellular senescence, there is a gradual decline in proliferative capacity, which can be related to the process of primary cells aging prior to and independently from senescence.

### 3.2 Increased cell soma

A property closely associated with cellular senescence is an enlargement of cell soma or “hypertrophy.” This property is a driving phenotype behind many senescence features such as an increase in organelle content, cytoplasm dilution and possibly also the permanent cell cycle arrest (Neurohr et al., 2019; Ogrodnik et al., 2019). Changes in cell size are inadvertently linked to cell proliferation; in standard tissue culture conditions, dividing cells must constantly increase their mass to compensate for any mass lost due to divisions. These two factors, division rate and growth rate, depend on one another and thus the cell size is impacted by a change in cell division rate and *vice versa* (Ginzberg et al., 2015). As a consequence, an acceleration of cell proliferation results in a smaller cell size whereas a slowdown of proliferation increases soma size (Angello et al., 1989; Ginzberg et al., 2015). It is thus not surprising that in the presence of factors which provide continuous growth stimulation (such as those used in fetal bovine serum), a permanent cell cycle arrest leads to an increase in cell size.

**FIGURE 2:**
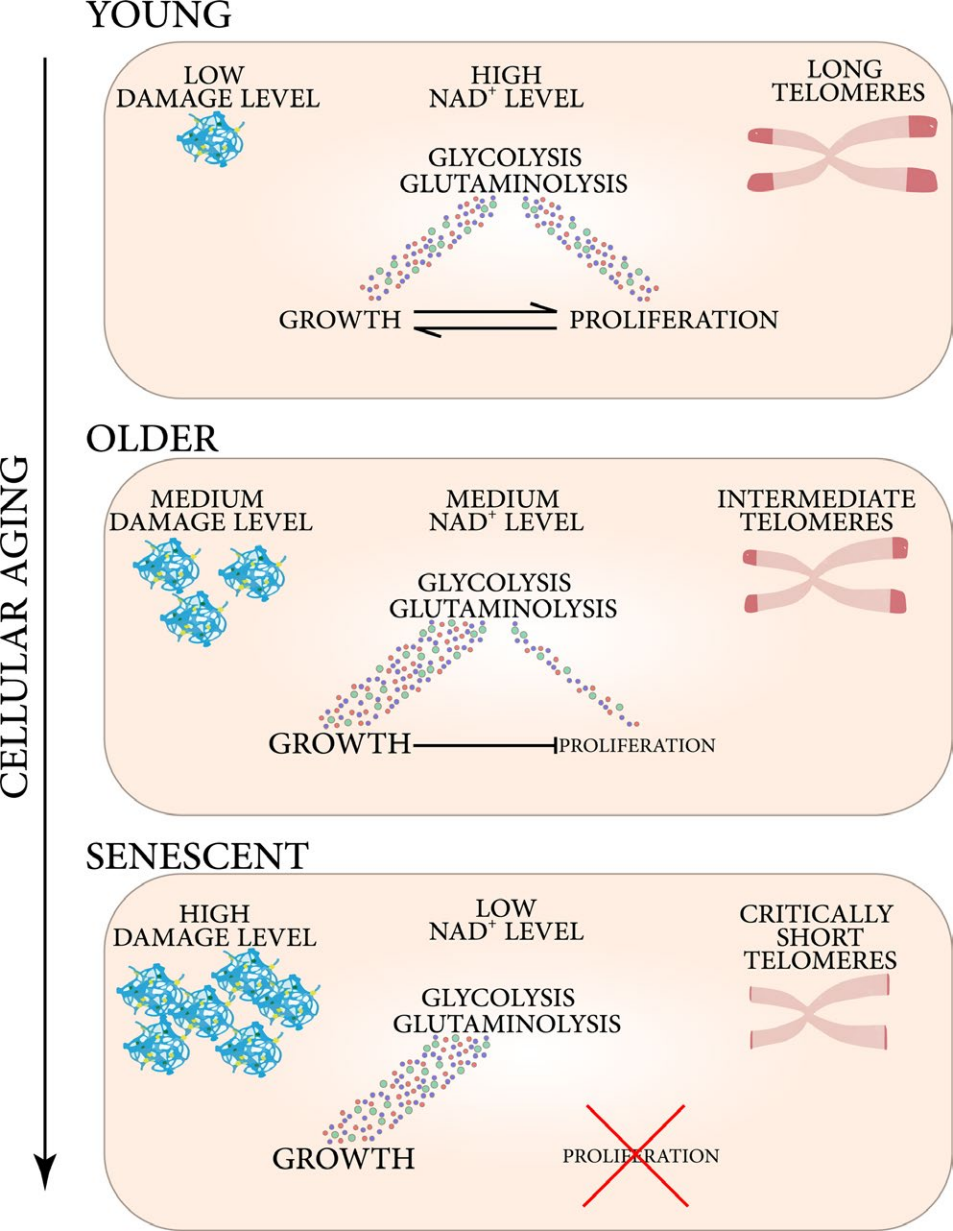
Phenotypical changes observed within aging cells. With progression of cellular aging, cells experience a gradual transition from the balanced state of coordinated growth and proliferation, to the state where soma growth dominates over proliferation. That occurs in parallel to metabolic shifts, reduction in NAD^+^, intracellular damage accumulation, and telomere shortening

Several studies reported that soma size of primary cells increases gradually during their replicative aging (Angello et al., 1987; Kim, Byun, et al., 2013; Nassrally et al., 2019; Pendergrass et al., 1989). Notably, this was a small and gradual increase in size of virtually all cells, rather than of a sub‐population (Angello et al., 1987; Nassrally et al., 2019). For example, using flow cytometry, Angello et al. showed that a cells’ replicative potential is inversely related to its early G1 volume (Angello et al., 1987). Alteration of cell size also impacts on the time of senescence induction: a classic experiment with maintaining cell culture at low density and at low serum concentration shows that an increase in cell volume by 50–100% leads to a significant loss of replicative potential (Angello et al., 1989). Similarly, a study by Pendergrass *et al*. showed that cells isolated from “giant” mice overexpressing a growth hormone show a decrease in replicative potential (Pendergrass et al., 1993). Another study showed a reverse correlation between the initial size of cells isolated from mice and their replicative potential (Yuan et al., 2006). Moreover, a short‐term inhibition of cell cycle with agents such as sodium butyrate, aphidicolin (Pendergrass et al., 1989) or palbociclib (Neurohr et al., 2019) increases cell size and reduces replicative potential of primary cells. Importantly, if cell growth during the temporary cell cycle inhibition is prevented by reducing the serum concentration, replicative potential of treated cells is restored (Neurohr et al., 2019). Similarly, live‐cell imaging revealed that late‐passage cells of increased soma size needed more time to initiate cell division (Nassrally et al., 2019). In summary, with progression of cellular aging there is a gradual increase in soma size occurring in parallel with an increase in time needed for cell division (Figure 2).

### 3.3 Metabolic shifts

Cell cycle progression requires coordination and high efficiency of many metabolic pathways to deliver sufficient influx of biomass and energy. An optimal experimental design needed to address the question on the effects of cellular aging on metabolism of primary cells would require metabolic profiling on a single‐cell level. Such approaches are currently available (Duncan et al., 2019; Emara et al., 2017), but they have not been applied in the context of aging studies. Certain studies were able, however, to use other approaches to characterize progression of metabolic changes occurring in primary cells during cellular aging. In a recent study, Yi *et al*. performed nuclear magnetic resonance (NMR) analysis of intracellular metabolites from five different time points (PD 4, 15, 31, 46, and 61) of human primary cells and observed a gradual decline in the levels of several metabolites including glutamate (product of glutaminolysis) and lactate (Yi et al., 2020). At the same time, the levels of glutamine increased, but the glucose levels did not change (Yi et al., 2020). These observations can already be related to the requirements for cell proliferation (reviewed in (Lunt & Vander Heiden, 2011)); however, in order to fully understand their impact, it would be necessary to examine the metabolic profile of senescent cells.

Both the import of glucose and the activity of many glycolytic enzymes are increased in senescent cells (Unterluggauer et al., 2008; Zwerschke et al., 2003). An increase in glycolysis is usually correlated with an increase in lactate production (Lunt & Vander Heiden, 2011). However, this correlation is not as straightforward in senescent cells. In contrast to young cells that produce more lactate when cultured in increasing concentration of glucose (Zwerschke et al., 2003), senescent cells do not show a linear relationship between glucose consumption and lactate production (Unterluggauer et al., 2008; Zwerschke et al., 2003). A remaining question is thus what happens with the products of glycolysis in senescent cells. In standard conditions, high glucose uptake combined with low lactate production would suggest that the carbon from glycolysis is used for oxidative phosphorylation (OXPHOS). Senescent cells show, however, a reduction in OXPHOS, lower content of ATP, and a higher AMP:ATP ratio (Unterluggauer et al., 2008; Wang et al., 2003; Zwerschke et al., 2003). If glucose is used neither for OXPHOS nor for lactate production, its metabolites could be utilized for an increase in biomass production. For example, glycolysis is one of the main sources of carbon for lipid precursors (Lunt & Vander Heiden, 2011), and senescent cells accumulate high quantities of lipids (Ogrodnik et al., 2017; Ogrodnik, Zhu, et al., 2019). A master regulator of protein synthesis and growth—the mammalian target of rapamycin (mTOR)—can be activated by intermediates of glycolysis and glutaminolysis with a certain level of redundancy between these pathways (Kim, Hoffman, et al., 2013). Senescent cells are characterized by activated mTOR, which is insensitive to amino acid and serum starvation (Carroll et al., 2017). This suggests that in senescent cells, glycolysis metabolites are sufficient to stimulate mTOR. In this context, findings from Yi *et al*. on a gradual decline in lactate and glutamine with an increase in glutamate in replicatively aging cells indicate that instead of using products of glycolysis and glutaminolysis for cell cycle progression, aging cells utilize them for the continuous stimulation of mTOR, protein and lipid synthesis, and cell growth. Overall, these observations indicate that cellular aging relates to a gradual shift from a metabolic profile utilizing glycolysis and glutaminolysis that orchestrates proliferation, to a metabolic profile, which uses glucose for cell maintenance and growth. Thus, it is possible that this metabolic shift occurring during cellular aging is related to a reduction in cell division rate and an increased cell soma size, therefore contributing to the induction of cellular senescence. In agreement with the observation that a gradual reduction in glutamine‐based metabolism impacts on the progression of replicative aging, it was shown that an inhibition of glutaminolysis is a potent driver of cellular senescence (Liao et al., 2019; Unterluggauer et al., 2008). For example, inhibition of glutamine‐metabolizing enzyme glutaminase (Unterluggauer et al., 2008), as well as inhibition of the rate‐limiting enzyme for glutamine metabolism in mitochondria, glutamate dehydrogenase (Liao et al., 2019), was shown to induce cellular senescence. Finally, inhibition of glutaminolysis was shown to induce apoptosis of senescent cells and to ameliorate various age‐associated disorders (Johmura et al., 2021).

Another metabolic feature of cellular aging observed by Yi et al. was a gradual decline in NAD^+^ concentration in cultures of cells approaching the Hayflick limit (Yi et al., 2020). This is in agreement with research on human primary and cancer cells, showing that conditions characterized by a reduction in NAD^+^ levels prevent cell proliferation (Chini et al., 2014; van der Veer et al., 2007; Zhang et al., 2002). NAD^+^ is generated in processes including OXPHOS and conversion of pyruvate to lactate (Verdin, 2015). Consistently with senescent cells showing a reduction in OXPHOS and lactate production, a reduction in NAD^+^/NADH ratio is observed in cells entering replicative and damage‐induced senescence (Nacarelli et al., 2019). Notably, oncogene‐induced senescence shows certain metabolic differences **(**Box 2). To signify the role of NAD^+^ reduction in the decline of replicative capacity, it was shown that supplementation of primary cells with NAD^+^ precursors increases replicative lifespan (Lim et al., 2006; Matuoka et al., 2001). Finally, replicative senescence was observed to be preceded by a decline in the expression and activity of an NAD^+^ recovering enzyme NAMPT: Its overexpression increased replicative lifespan (van der Veer et al., 2007), while knockdown or inhibition reduced it (Nacarelli et al., 2019). As a reduction in cellular NAD^+^ content does not interfere with cell soma growth (Chini et al., 2014; van der Veer et al., 2007), a gradual NAD^+^ depletion during cellular aging is a likely contributor to a transition from proliferation to biomass production (i.e., an increase in soma size) (Figure 2).

### 3.4 Telomere shortening

The dominant theory explaining the induction of replicative senescence describes a progressive replication‐dependent shortening of telomeres—sequences found at the end of chromosomes (d'Adda di Fagagna, 2008; d'Adda di Fagagna et al., 2003). Upon reaching a certain “critical” length, telomeres are recognized as double‐strand breaks (DSBs), leading to the activation of the DNA damage response (DDR) and cell cycle arrest (d'Adda di Fagagna, 2008; d'Adda di Fagagna et al., 2003). DSBs can also occur at telomeres independently of their length, in conditions of genotoxic stress or even spontaneously (Doksani & de Lange, 2016; Fumagalli et al., 2012; Hewitt et al., 2012; Mao et al., 2016). Telomere‐associated DSBs can be repaired in proliferating cells (Doksani & de Lange, 2016; Mao et al., 2016), but not in non‐dividing (e.g., post‐mitotic) or cell cycle‐arrested cells (Fumagalli et al., 2012; Hewitt et al., 2012). While this type of damage is detectable in young, primary cells (Fumagalli et al., 2012; Hewitt et al., 2012), it has not been shown whether telomeric DSBs at the levels found in young cells may have any phenotypic consequence or whether this type of damage increases in frequency during cellular aging.

There are numerous pieces of evidence supporting the role of telomere shortening in the induction of replicative senescence, including a decrease in average telomere length over time in cultured cells, overexpression of telomerase (an enzyme responsible for increasing telomere length) circumventing senescence (reviewed in (Bernadotte et al., 2016)) and even an inverse correlation between telomerase activity and senescence *in vivo* (Cheng et al., 2019). It needs to be highlighted, however, that not all the evidence unambiguously supports the hypothesis of telomere‐shortening‐driven senescence. In fact, it has been questioned if telomerase‐induced immortality is strictly linked to telomere lengthening **(**Box 3). A gradual increase in cell size and duration of cell cycle prior to replicative senescence can be linked to the process of telomere shortening. For example, in a study done by Nassrally et al., the authors showed that an increase in cell size and an extension of cycle time progresses in parallel to an increase in population doublings and thus also in parallel to a reduction in telomere length (Nassrally et al., 2019). These effects were absent in cells overexpressing telomerase indicating that canonical or non‐telomeric functions of telomerase (Box 3) are sufficient to prevent certain aspects of cellular aging (Nassrally et al., 2019). A remaining question concerns the mechanisms that could explain the effect of an intermediate telomere length on the features of cellular aging. Despite telomere shortening being a gradual process, it is generally assumed that only critically short telomeres can affect the process of replicative aging (Bernadotte et al., 2016). In other words, if an intermediate telomere length has no functional output, the phenotype of cells based on their telomere length would be binary: senescent cells have “critically short” telomeres, while any cell with longer than “critically short” telomeres is a young cell. An intermediate length of telomeres eliciting a phenotypic effect could indicate aging prior to cellular senescence.

One possibility is that telomere shortening causes proteins canonically binding telomeres to change their location and function. Telomere‐binding proteins or “shelterins” stabilize the structure of chromosome ends and protect them from recognition by DDR‐related proteins (de Lange, 2018). However, the role of certain shelterins goes beyond telomeric protection and they have been shown to regulate gene expression as well as to interact with cytoplasmic proteins regulating their activity (Lian et al., 2013; Martinez et al., 2010; Teo et al., 2010). For example, the shelterin protein Rap1 was discovered to translocate from telomeres to extratelomeric binding sites, where it regulates gene expression (Martinez et al., 2010), as well as to the cytoplasm where it activates NF‐kB via binding to the NF‐kB negative regulator IKK (Lian et al., 2013; Teo et al., 2010). The NF‐kB pathway controls inflammation and influences cell growth and apoptosis (Inoue et al., 2007). In yeast, the amount of Rap1 in the cytoplasm strictly depends on telomere length (Platt et al., 2013). This relationship has not been established in mammalian models; nevertheless, certain evidence exists indicating that Rap1 re‐localizes from telomeres in a length‐dependent manner, for example in telomerase‐deficient mice and cells (Martinez et al., 2016). Finally, oxidative stress, known to induce senescence and to shorten telomeres (Richter & von Zglinicki, 2007; von Zglinicki, 2002), also reduces Rap1 levels in the nucleus (Swanson et al., 2016).

Another candidate protein which could be mediating the effects of telomere shortening in a gradual manner is a Rap1‐binding partner, the shelterin TRF2. With gradually decreasing telomere length, TRF2 increases its occupancy on non‐telomeric chromatin regions, where it regulates epigenetic modifications and transcription (Mukherjee et al., 2018). In addition, the extratelomeric activity of TRF2 has been shown to contribute to angiogenesis (El Mai et al., 2014; Zizza et al., 2019) and mitochondria function in muscle (Robin et al., 2020). Similarly to Rap1, TRF2 translocates from telomeres to non‐telomeric chromatin upon DNA damage (Bradshaw et al., 2005) and in senescence (Mitchell & Zhu, 2014). Notably, Rap1 binds to telomeres solely through TRF2 (Celli & de Lange, 2005; Takai et al., 2010) suggesting that with decreasing telomere length, the dissociation of TRF2 from telomeres could also drive the relocation of Rap1. Overall, these results suggest that changes in telomere length can affect and coordinate cell functions not only when telomeres become critically short or damaged, but also in a gradual manner matching the progression of cellular aging.

### 3.5 Intracellular damage

The term “damage,” even if narrowed down to “intracellular damage,” is difficult to define as it encompasses every modification of a biomolecule that negatively affects its function or stability (Gladyshev, 2012, 2013; Ogrodnik et al., 2019). As each biomolecule can be damaged in multiple ways, damage forms are far more numerous than the systems designated to cope with them. Therefore, only the damage forms which are the most toxic and/or have the most immediate effects have their dedicated detection and repair systems. Other forms of damage are challenging not only for cellular systems, but even for researchers as the currently available detection methods are limited and little data have been collected. The literature of senescence is focused mostly on damage forms which are the simplest to detect, while the presence of other damage forms can be only speculated from their causes or consequences. Thus, this part of the article is focused on two of the most common senescence‐associated damage forms: DNA double‐strand breaks and lipofuscin.

Double‐strand breaks (DSBs) are considered one of the most toxic forms of DNA damage, and their immediate consequences include cell cycle arrest and apoptosis (reviewed in (White & Vijg, 2016)). DSBs are constantly being generated by environmental factors, cell metabolism and changes in DNA topology (Malaquin et al., 2015). There have been several reports suggesting a gradual increase in the frequency of foci associated with DSB sites in aging of primary cells (Fumagalli et al., 2014; Pustovalova et al., 2016; Rodier et al., 2009; Sedelnikova et al., 2004; Suzuki et al., 2012). An interesting aspect of the increase in frequency of DSBs during replicative aging is that it is often reported to be non‐linear (Pustovalova et al., 2016; Rodier et al., 2009). For example, Rodier et al. showed that the frequency of cells positive for DSBs increases linearly until mid‐age when it plateaus, reaching a value that is roughly equal to the frequency of DSBs‐positive cells in a population of senescent cells (Rodier et al., 2009). In other words, the frequency of DSB‐bearing cells during replicative aging reaches its maximum while there is still a large fraction of cells capable of proliferating. This and other research groups reported that although the presence of DSBs reduces the chances for cell division, primary cells are still capable of dividing while bearing even several DSB foci (Nassrally et al., 2019; Rodier et al., 2009). What processes could be responsible for an increase in the frequency of DSBs throughout replicative aging? In cell culture conditions, one of the strongest drivers of DSBs is replication and especially, so‐called “replication stress” (Gelot et al., 2015; Lopez‐Contreras & Fernandez‐Capetillo, 2010; Zorin et al., 2019). Increase in cell cycle duration as well as the abovementioned changes in metabolism of primary cells is among the main inducers of replication stress (Magdalou et al., 2014). Consistently, hallmarks of replication stress have been shown for primary cells approaching Hayflick limit (Rivera‐Mulia et al., 2018).

In a proliferating cell, the amount of DSBs which surpass its repair capacities results in an induction of cell senescence or apoptosis. One outstanding question is whether DSBs could affect cell viability outside of cell cycle arrest, cellular senescence or apoptosis. DSBs, even if repaired, often result in detrimental changes in DNA structure and sequence. For example, DSBs at sub‐telomeric regions lead to deletions (Mao et al., 2016; Miller et al., 2011), at telomeres DSBs correlate with accelerated shortening (Berardinelli et al., 2013; De Vitis et al., 2019; Doksani, 2019), and in the genome‐wide DNA, they lead to mutations and chromosomal instabilities (Dolle & Vijg, 2002; Lieber & Karanjawala, 2004; White & Vijg, 2016). Telomere shortening and chromosomal instabilities are well‐established drivers of senescence (Bernadotte et al., 2016; Busuttil et al., 2004). Although it is not known how the deletions or mutations contribute to senescence, an increase in an average number of mutations has been observed during replicative aging (Busuttil et al., 2003; Caliri et al., 2020). Notably, although DSBs affect genome integrity only locally (i.e., at the site where the break occurs), even such insults could lead to global consequences; for example, damage‐induced shortening of a small fraction of telomeres can lead to cell cycle arrest (Zou et al., 2004). Overall, a gradual increase in DSBs in aging primary cells is likely to lead to phenotypic consequences prior to senescence induction.

Lipofuscin is an autofluorescent intracellular deposit consisting of oxidized and modified lipids and proteins (Terman & Brunk, 2004). Levels of lipofuscin increase gradually with passage number of several types of primary cells (Ksiazek et al., 2009; Sitte et al., 2001). Similarly to lipofuscin itself, the levels of its components, such as carbonylated, glycated, and lipid peroxidation‐modified proteins, have been shown to gradually increase during replicative aging of human primary cells (Baraibar et al., 2016). Importantly, approaches using single‐cell spectroscopy and flow cytometry have revealed that the increase in lipofuscin in aging primary cells is not driven by a large increase in a small population of prematurely senescent cells, but rather by a gradual increase in the majority of cells (Eberhardt et al., 2017). Similarly to several other senescence markers (Ogrodnik, Salmonowicz, & Gladyshev, 2019), lipofuscin can accumulate in primary cells over time, even if these cells are not dividing, that is, post‐mitotic or quiescent (Burke & Skumatz, 1998; Eberhardt et al., 2018; Moreno‐Blas et al., 2019). It is intriguing to speculate that lipofuscin may derive from an imperfect cellular metabolism, making its accumulation dependent on metabolic rate rather than proliferation.

## 4 CELLULAR SENESCENCE AND CELLULAR AGING *IN VIVO*

The research focused on the characterization of cellular senescence *in vivo* occurred on a relatively low scale through the 90’s and 00’s; however, during the second decade of the 21st century, the number of research articles focusing on this subject has increased exponentially. This shift is owed not only to the development of new senescence markers, a transition from immunohistochemistry (IHC) to immunohistofluorescence (IHF) for more precise senescence quantification *in situ*, but also due to the generation of senescence reporter mouse models (Baker et al., 2011; Demaria et al., 2014; Liu, Souroullas, et al., 2019), as well as methods to eliminate senescent cells in living mice (Chang et al., 2016; Yosef et al., 2016; Zhu et al., 2015). Despite these advances, quantification of senescence in animal tissues is still challenging and the estimates of the precise number of senescent cells can vary more than 10‐fold between laboratories, despite the use of similar methods in similar conditions (Table 1). The majority of methods commonly used to detect senescent cells, such as IHC/IHF, flow cytometry, and real‐time PCR, allow for the assessment of only a single marker per experiment, which complicates drawing comparisons between markers and experimental conditions. In the previous parts of this article, it was shown that cells positive for certain senescence markers might be advanced in cellular aging, but not necessarily senescent. Introduction of methods for assessment of multiple markers at the single‐cell level such as single‐cell RNA sequencing (Tabula Muris, 2020; Tang et al., 2019) or cytometry by the time of flight (Ogrodnik, Zhu, et al., 2019; Palmer et al., 2019) might refine senescence assessment *in vivo* in the future.

**TABLE 1:** Quantification of percentage of senescent cells in old (18–32 m), wild‐type, C57Bl/6 mice. DSBs is “Double‐strand breaks”; SA‐β‐gal is “senescence‐associated‐beta‐galactosidase”; EM is “Electron microscopy”; FC is “Flow cytometry”; scRNA‐seq is “single‐cell RNA sequencing”

Percentage of senescent cells in old (18–32 m), wild‐type, C57Bl/6 mice			
Article	Senescence marker	Tissue (cell type if specified)	% of positive cells
Jurk et al. (2012)	DSBs	Brain (Purkinje neurons)	~40%
	Lipid peroxidation	Brain (Purkinje neurons)	~80%
	colorimetric SA‐β‐gal	Brain (Purkinje neurons)	~60%
	DSBs	Brain (cortical neurons)	~40%
	Lipid peroxidation	Brain (cortical neurons)	~20%
	colorimetric SA‐β‐gal	Brain (cortical neurons)	~80%
Hewitt et al. (2012)	Telomere‐associated DSBs	Small intestine (enterocytes)	30–40%
	Telomere‐associated DSBs	Liver (hepatocytes)	15–25%
Zhu et al. (2015)	colorimetric SA‐β‐gal	Inguinal Fat	~8%
Xu et al. (2015)	colorimetric SA‐β‐gal	Visceral Fat	~20%
Birch et al. (2015)	Telomere‐associated DSBs	Lungs	~20%
Biran et al. (2017)	FC‐based SA‐β‐gal	Inguinal Fat (stromal cells)	~12%
	FC‐based SA‐β‐gal	Spleen (stromal cells)	~4%
	FACS‐based SA‐β‐gal	Small intestine (stromal cells)	~3%
Baker et al. (2016)	EM‐based SA‐β‐gal	Visceral Fat	~2%
	EM‐based SA‐β‐gal	Kidney	~2%
	EM‐based SA‐β‐gal	Heart	~10%
	FC‐based p16 (GFP)	Inguinal Fat	~5%
Ogrodnik et al. (2017)	DSBs	Liver (hepatocytes)	~15%
	Telomere‐associated DSBs	Liver (hepatocytes)	~15%
Anderson et al. (2019)	Telomere‐associated DSBs	Heart (cardiomyocytes)	50–70%
	colorimetric SA‐β‐gal	Heart (cardiomyocytes)	~4%
	Lipid peroxidation	Heart (cardiomyocytes)	~70%
	DNA oxidation	Heart (cardiomyocytes)	~20%
Liu, Souroullas, et al. (2019))	FC‐based p16 (tdTom)	Pancreas	~3%
	FC‐based p16 (tdTom)	Inguinal Fat (progenitor cells)	~6%
	FC‐based p16 (tdTom)	Cartilage	~6%
Iske et al. (2020)	p16/p21 (antibody)	Skin	~8%
	p16/p21 (antibody)	Heart	9–10%
	colorimetric SA‐β‐gal	Kidney	~10%
Tabula Muris (2020)	scRNA‐seq‐based p16	Multiple tissues	2–3%^a^

^a^ Converted from fraction.

**FIGURE 3:**
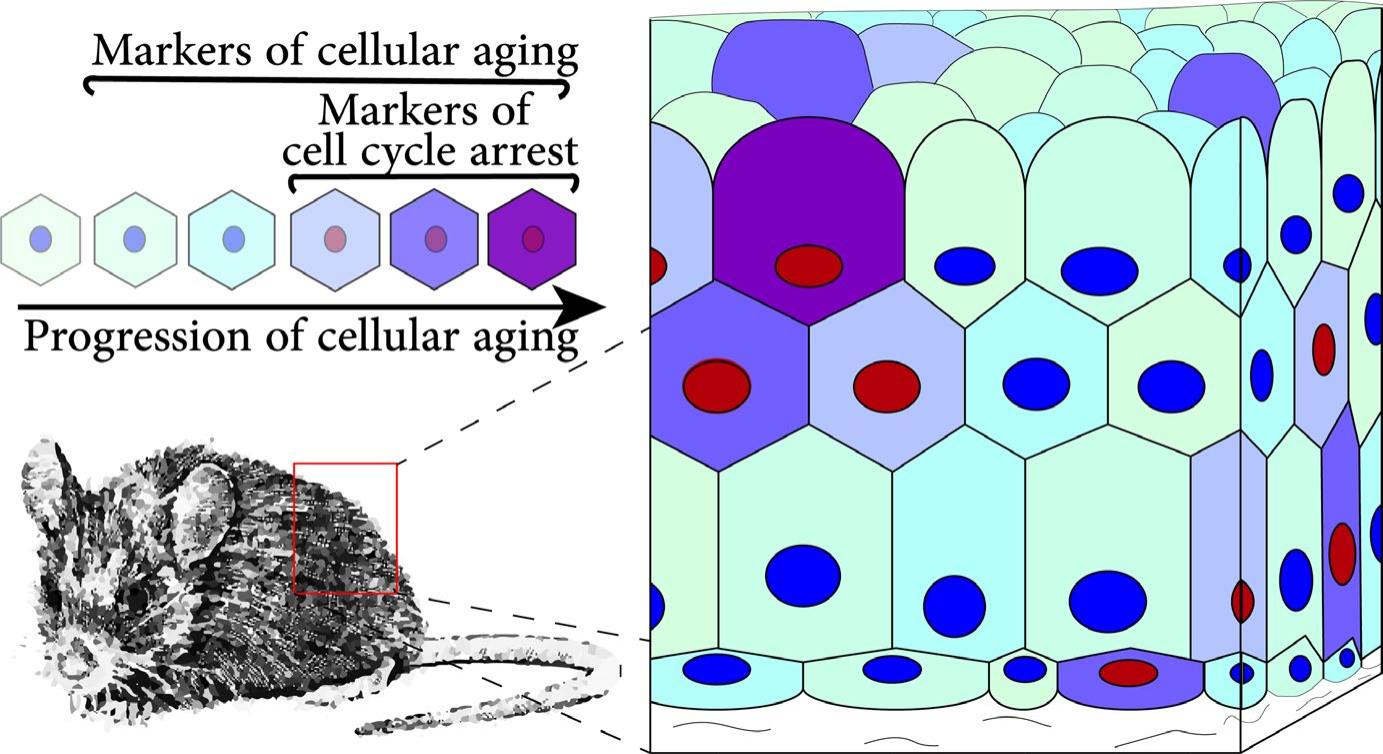
The concept of cellular aging *in vivo*. Cells found in tissues of aging animals are at different stages of cellular aging, thus showing markers associated with cellular senescence before the establishment of the cell cycle arrest. In addition, senescent cells *in vivo* might show different levels of senescence markers as cellular aging progresses even after senescence induction and cells continue changing over time until death

Another difficulty with assessment of cellular senescence *in vivo* derives from a difference in cellular landscapes between *in vitro* and *in vivo* conditions. In addition to the high complexity of spatial and functional arrangements of cells *in vivo*, even in models with high senescence content, tissues contain a mixture of cells at different stages of cellular aging (Figure 3). This contrasts the classic experimental design of senescence assessment *in vitro*, where comparisons between pure populations of young and senescent cells are made. In other words, the concept proposed here is that senescence detection *in vivo* can be affected by the presence of cells advanced in cellular aging, thus positive for certain senescence markers, but which are not yet senescent (Figure 3). Finally, it has bee hypothesized that during aging certain cell types do not become arrested in cell cycle, and would not undergo senescence transformation (Sun et al., 2014), but can advance in cellular aging accumulating markers of damage and cellular dysfunctions (Hinge et al., 2020, Wang et al., 2012).

### 4.1 Binary markers of cellular senescence in vivo

In theory, there are markers which should define the binary commitment of cellular senescence, including those associated with permanent cell cycle arrest and chromatin modifications. In practical terms, however, such markers are challenging to be used. In contrast to *in vitro* conditions, where the basal state of cells is continuous proliferation, the majority of cells *in vivo* divides rarely. Thus, if a cell *in vitro* is found to be negative for a marker of proliferation, such as Ki67 or PCNA, it is likely this cell is arrested in cell cycle and possibly senescent. However, a cell negative for markers of proliferation *in vivo* is more likely to be quiescent or post‐mitotic, and its phenotype unrelated to cellular senescence. Another approach to detect cell cycle arrested cells involves assessing the expression levels of cell cycle inhibitors such as p16 (CDKN2a) or p21 (CDKN1a). This method allows for very efficient detection of senescent fibroblasts *in vitro*; however, *in vivo* both these proteins have been shown to be expressed in a transient manner in processes unrelated to cellular senescence such as terminal differentiation (Aix et al., 2016; Puente et al., 2014; Tane et al., 2014) and macrophage activation (Hall et al., 2016, 2017). Detection of senescence‐specific chromatin alterations bears a promise of high specificity, however, on a single‐cell level is methodologically challenging and has not been optimized for *in vivo* conditions. Nevertheless, development of such a marker could potentially enable more precise detection of senescent cells *in vivo*.

### 4.2 Markers of cellular aging in vivo

In this section, the distribution patterns of senescence markers *in vivo* will be discussed. Specifically, it will be considered whether certain senescence features such as lipofuscin accumulation, DNA, and oxidative damage tend to be present in a small fraction of cells and represent senescence or in a higher fraction of cells and represent cellular aging. The majority of published articles does not present data distribution of measurements from individual cells, but rather shows results as an average, which is derived from measurements of all the cells per sample. Similarly to the results of *in vitro* studies, such a way of data presentation prevents drawing conclusions on what the variance level per sample actually is. In other words, information on whether a change in the level of a senescence marker between two samples comes from a change in a fraction of highly positive cells (i.e., senescent) or from a smaller change in all cells (i.e., advancing in cellular aging) cannot be extracted from a comparison between average levels of the marker per sample. This question can be, however, approached from a different angle.

The paradigm established for cellular senescence *in vivo* is tightly linked to an assumption that only a small fraction of cells becomes senescent. This assumption comes from the reasoning that senescent cells are highly dysfunctional and an organ with a high number of senescent cells would not be able to maintain its proper function, causing death of an animal; moreover, treatments killing senescent cells could prove potentially detrimental if they targeted a large fraction of an organ's parenchyma. For example, studies measuring the number of cells positive for the senescence marker senescence‐associated‐beta‐galactosidase (SA‐β‐gal) report that the fraction of senescent cells is lower than 2% in visceral fat and kidneys of old mice (Baker et al., 2016). Similarly, a recent study utilizing single‐cell transcriptomics reported that the number of p16‐positive cells in organs of old mice is 2–3% (Tabula Muris, 2020). In contrast, many studies reported significantly higher frequencies of senescent cells, even if only reports concerning naturally aging mice are considered (Table 1). For example, measurements of senescence markers such as lipid peroxidation and DSBs showed that 40–80% of Purkinje and 20–40% of cortical neurons become positive in old mice (Jurk et al., 2012). Similarly, 50–70% of cardiomyocytes (Anderson et al., 2019) and 30–40% of enterocytes (Hewitt et al., 2012) were reported positive for senescence markers such as telomere‐associated DSBs and lipid peroxidation. Collectively, estimates from 12 studies on the percentage of senescent cells in a variety of tissues from old (18–32 m), wild‐type, C57Bl/6 mice show that markers of senescence SA‐β‐gal and p16 are usually detected in ≤10% of cells, while markers associated with damage including lipid peroxidation, DSBs, and telomere‐associated DSBs are predominantly detected in >20% (and up to 80%) of cells (Table 1). Thus, datasets showing a high number of cells positive for senescence markers likely also include cells which are still prior to cell cycle arrest and senescence induction. These cells can be considered advanced in the process of cellular aging, but not yet senescent.

Similar conclusions can also be drawn from studies using thresholding of continuously distributed senescence markers to separate senescent and non‐senescent cells as a method to show changes in the number of senescent cells. As it is not known what level of senescence markers (such as DNA damage) is needed to trigger senescence *in vivo*, attempts have been made to set an arbitrary threshold beyond which cells are considered to be senescent. For example, in Ogrodnik et al., the minimum number of telomere‐associated DSBs considered to define hepatocyte senescence is 3 (Ogrodnik et al., 2017). If livers of old animals consisted of a mixture of young and senescent cells, without any in‐between stages (i.e., cellular aging) there would be no cells showing levels of markers below the threshold (i.e., cells with 0 < x < 3 telomere‐associated DSBs). However, cells showing 1 or 2 telomere‐associated DSBs are not only present, but clearly accumulate during aging (Ogrodnik et al., 2017).

Importantly to the *in vivo* context, cellular aging might progress even if cells are not dividing i.e. are quiescent or post‐mitotic cells. The process of cellular aging in quiescent cells can be referred to as “deepening quiescence” (Fujimaki & Yao, 2020) and described as a gradual reduction in cell capacity for proliferation resumption when triggered by growth signals. Consistently, cells which are maintained in a quiescent state accumulate lipofuscin (Eberhardt et al., 2018), show progressive lysosomal dysfunction (Fujimaki et al., 2019) as well as gradual accumulation of DNA double‐ (Marthandan et al., 2014) and single‐strand breaks (Sitte et al., 1998). Similarly, senescence markers are acquired over time by post‐mitotic cells such as neurons, cardiomyocytes, adipocytes, and osteocytes *in vitro* and *in vivo* (Anderson et al., 2019; Farr et al., 2016; Jurk et al., 2012; Minamino et al., 2009; Ogrodnik, Zhu, et al., 2019; Xu et al., 2015). Although it is a truism that cells age when their host (an organism) does, in the context of senescence markers *in vivo* it is important to stress that the process of cellular aging, which shares markers with cellular senescence, progresses in virtually all cells. The result is a fraction of cells that may bear a higher damage load yet are not in the state of cell cycle arrest (Figure 3). This is of primary importance for clinical trials measuring the effects of anti‐senescence interventions on markers of senescence as it is not known whether senolytic drugs differentially affect cells considered to be advanced in cellular aging than cells in established senescence.

### 4.3 Specificity of senescence markers in vivo in the context of cellular aging

The experimental evidence presented in the previous paragraphs suggests that markers of senescence associated with a variety of damage forms are present in a higher number of cells than markers of senescence associated with cell cycle arrest or lysosomal dysfunction (SA‐β‐gal). These discrepancies can be explained by separating senescence markers into causes and consequences of senescence induction. In this context, markers such as oxidative and DNA damage (as well as several others described above in the context of *in vitro* applications) can lead to senescence and thus can be present in cells prior to senescence induction, while upon entering the state of senescence they would remain the same or even increase. In contrast, markers such as p16 and SA‐β‐gal are associated with later stages of senescence establishment (sometimes referred to as “deep senescence”) and would not be expected to characterize cells prior to senescence induction. This consideration suggests that other markers, such as an increase in cell size, a slowdown of proliferation, and damage should be associated more closely with cellular aging rather than with cellular senescence.

Based on this evidence, we suggest a definition of the markers of cellular aging—such markers (I) can be present in proliferating cells, (II) accumulate in a gradual manner, (III) can have phenotypic effects prior to and/or independent from the senescence context, and (IV) can be directly responsible for the induction of cellular senescence. However, the hypothesis that certain senescence markers are actually more suitable to define cellular aging than cellular senescence does not invalidate them as senescence markers. First, no marker of senescence is of sufficient specificity. Damage‐related markers might be present in processes beyond senescence such as cellular aging, but also p16 and SA‐β‐gal are present in transiently activated immune cells (Hall et al., 2016, 2017) or in growth‐stimulated quiescent and confluent cells (Leontieva & Blagosklonny, 2014; Severino et al., 2000). Second, in contrast to p16 and SA‐β‐gal, which are sometimes associated with reversibility (Hall et al., 2016, 2017; Leontieva & Blagosklonny, 2014; Severino et al., 2000), markers of damage can be considered less reversible, especially in the *in vivo* context. This is due to reduced repairability and/or removability of damage types such as lipofuscin (Terman & Brunk, 2004) and telomere‐associated DSBs in non‐dividing cells (Fumagalli et al., 2012; Hewitt et al., 2012). Finally, it is also worth noting that senolytic interventions reduce not only p16‐ and SA‐β‐gal‐positive cells (as in (Baker et al., ,^2011^, ^2016^)), but also cells positive for DNA and lipid damage (as in (Anderson et al., 2019; Ogrodnik et al., 2017; Ogrodnik, Zhu, et al., 2019)), suggesting that all these markers have indeed a certain level of suitability for indication of senescence prevalence *in vivo*.

Thus, a multi‐marker approach for cellular senescence characterization has a chance not only to provide more reliable values for senescence abundance, but in case of a disparity between levels of markers, can also indicate its origin. Hypothetically, in a disease‐afflicted organ, detection of high p16 and low damage levels could indicate that the phenotype is driven by activation of immune cells, while detection of low p16 and high damage levels could indicate a dominant role of cellular aging. For example, recent research showed that the number of p16‐positive cells plateaus in mice of very old age (Tabula Muris, 2020) (as predicted in (Ogrodnik, Salmonowicz, Jurk, et al., 2019)), while damage markers increase throughout the whole murine lifespan, even in the oldest mice (Birch et al., 2015; Hewitt et al., 2012). This observation on the differences in kinetics of markers of cellular aging and senescence during animal aging could lead to the development of therapeutic applications against age‐related diseases. For the development of such applications, however, more research on characterization of senescence markers on a single‐cell level is necessary.

## 5 CELLULAR AGING AFTER CELLULAR SENESCENCE INDUCTION

Thus far, the emphasis has been predominantly on how the concept of cellular aging relates to the phenotype of primary cells prior to the induction of cellular senescence. The following section of this article will describe the role of cellular aging for the last stage of cellular life according to Hayflick (Hayflick, 1991), namely for the period from the onset of cellular senescence until cell death (Figure 1a).

The final stage of cellular aging, which is characterized by an increase in cell death and a gradual degeneration of primary cells population, has not been investigated in much detail. Despite high viability of senescent cells under *in vitro* conditions that allows them to avoid death for months to years after senescence induction (Fumagalli et al., 2014; Sitte et al., 2000; von Zglinicki et al., 1995), most research has focused on the phenotype of cellular senescence only days to weeks after the induction. Cellular aging does not stop with the induction of cellular senescence and instead progresses until cell death. Several recent studies have described the changes occurring in cells after senescence induction (Hoare et al., 2016; Martinez‐Zamudio et al., 2020; Teo et al., 2019), it is, however, unclear what the kinetics of senescence markers after senescence induction are. On one hand, senescent cells show an acceleration in accumulation rate of many damage forms (reviewed in (Ogrodnik, Salmonowicz, & Gladyshev, 2019)). On the other hand, a study by Fumagalli et al., which followed the kinetics of DNA damage level in senescent cells for up to 3 months after the induction, showed that the average levels of certain damage forms in senescent cells might be constant or even decrease in the months following senescence induction (Fumagalli et al., 2014). This might be due to the higher death rate of senescent cells that bear the highest damage load, and for some types of primary cells, which are characterized by a high risk of cell death after senescence induction, the damage level might remain constant or even decline over time (Fumagalli et al., 2014). Finally, studying progression of cellular aging beyond induction of cellular senescence might allow for identification of new markers and phenotypes of old cells. For example, a study by De Cecco et al. showed that one of the features of genome degeneration, an activation of retrotransposons, is initiated only several weeks after senescence induction and progresses in a gradual fashion from that point on (De Cecco et al., 2019). A detailed examination of the progression of cellular aging *in vivo* should be of high focus for future studies as it is likely that senescent cells observed during aging initially senesced a long time ago, and are advanced in the process of cellular aging, nearing death. In summary, the concept of cellular aging implements a description of how cells gradually change from their youthful phenotype, through replicative aging, senescence induction and toward an unavoidable death, therefore providing novel opportunities for characterization of cellular senescence *in vivo*.

## 6 CONCLUSIONS AND OPEN QUESTIONS

One of the most profound advancements in the science of aging is the expansion of the field of cellular senescence to the stage of *in vivo* research. Discoveries concerning the role of senescent cells in animal physiology and pathology, as well as the introduction of senolytic interventions to target senescence arise as a great promise to treat age‐related diseases and ultimately, to prolong human healthspan. The conceptual framework on cellular senescence, especially for *in vivo* conditions is, however, incomplete and an increasing number of studies report conflicting results and anomalies.

Introduction of the concept of cellular aging refines the view on cellular senescence providing explanations for the presence of anomalies found *in vitro* and *in vivo*, resolving discrepancies in senescence quantifications between studies and rationalizing evidence on the high frequency of cells positive for markers associated with damage *in vivo*.

However, research on this subject is still in its infancy. For example, many markers associated with cellular aging *in vitro*, such as a gradual increase in cell soma, metabolic shifts and a slowdown of cell proliferation have not been characterized on the single‐cell level *in vivo*. Similarly, the relationship between cellular aging and cellular senescence is almost completely unknown for the *in vivo* conditions and questions such as: “How long can cells persist and continue to age after senescence induction?”, “Are there any phenotypic differences between cells short‐ and long‐term after senescence induction?” and “What is the role of non‐senescent cells, however, advanced in cellular aging in age‐related diseases and the process of aging?” are challenging us to be addressed in the future.

BOX 1 **Bystander effect and cellular aging**

The bystander effect of senescent cells negatively affects non‐senescent cells via reactive oxygen species (ROS) (Nelson et al., 2012, 2018) and SASP (Acosta et al., 2013). Studies showed that a paracrine effect of senescent cells can damage DNA (Nelson et al., ,^2012^, ^2018^), and even induce a permanent cell cycle arrest (Acosta et al., 2013). The bystander effect provides an alternative explanation of cellular aging, namely that even a small fraction of senescent cells, which arises earlier/prematurely in the replicative lifespan, increases levels of senescence markers in young cells. It should be noted, however, that in the aforementioned studies, the results were obtained by co‐culturing young cells with a high number of damage‐ or oncogene‐induced senescent cells, which are known to have a very prominent secretory phenotype (Nacarelli et al., 2019). Such artificial conditions are unlikely to exist within the population of replicatively aging cells or *in vivo*. Moreover, these studies did not report on many of the senescence markers associated with cellular aging such as changes in cell size or division time. In addition, the bystander effect has been often reported to stimulate, rather than inhibit cell proliferation, for example of epithelial and cancer cells (Bavik et al., 2006; Guan et al., 2017; Krtolica et al., 2001). Likewise, a study by Nassrally et al. demonstrated that even without an active p16, which executes induction of premature senescence, cells show markers of cellular aging, such as an increase in soma size, an increase in the risk of spontaneous cell death and a slowdown of proliferation (Nassrally et al., 2019). Finally, even if the results of the single‐cell approaches arose from the bystander effect rather than the cell‐autonomous changes, it is still an indication that there are aging‐related changes in non‐senescent cells matching the concept of cellular aging. Overall, these results suggest that the bystander effect is a possible contributor to the process of cellular aging, however, it does not undermine the concept.

BOX 2 **An anti‐warburg effect characterizes oncogene‐induced senescence**

Oncogene‐induced senescence (OIS) is driven by a high level or activity of oncogenic proteins such as RAS or BRAF^V600E^ (Di Micco et al., 2006; Michaloglou et al., 2005). Induction of OIS changes cellular metabolism; however, there are major differences between OIS and other types of senescence. For example, while replicative senescence increases glycolysis (Unterluggauer et al., 2008; Zwerschke et al., 2003), OIS reduces expression of genes related to glycolysis and elevates expression of genes involved in mitochondrial respiration (Li et al., 2013). Moreover, in contrast to replicative senescence, which shows a reduction in NAD^+^ recovering enzyme NAMPT, OIS shows an increase in level and activity of this enzyme (Nacarelli et al., 2019). These changes, together with an increase in level and activity of pyruvate dehydrogenase (PDH) (a gatekeeping enzyme linking glycolysis to the tricarboxylic acid cycle) (Kaplon et al., 2013), lead to an upregulation of OXPHOS in OIS (Kaplon et al., 2013; Nacarelli et al., 2019) and an increase in NAD^+^/NADH ratio (Nacarelli et al., 2019). The shift from glycolysis to OXPHOS suggests that the phenotype of OIS cells is, in many respects, opposite to the phenotype of cancer cells and was thus termed the “anti‐Warburg effect” (Li et al., 2013). High NAD^+^ content of OIS cells is also believed to result in the highest SASP level of all the types of cellular senescence (Nacarelli et al., 2019).

BOX 3 **Telomere‐independent functions of telomerase and cellular aging**

Telomerase is best known for increasing the length of telomeres, repetitive DNA sequences at the termini of chromosomes (Blackburn et al., 2006). As overexpression of telomerase is often sufficient to prevent telomere‐shortening‐induced replicative senescence (Bernadotte et al., 2016), it was assumed that the main action of telomerase to counteract senescence is via telomere lengthening. However, over the years, the role of telomerase has been shown also in telomere‐independent contexts. In a proof‐of‐concept study by Sun et al., the authors found that the re‐introduction of telomerase to cells from telomerase‐deficient (the 26th generation of mTert^+/−^) mice, which have extremely short telomeres, is sufficient to increase their replicative lifespan and to decrease the rate of malignant transformation (Sun et al., 2019). Importantly, these changes occurred without a significant increase in telomere length (Sun et al., 2019). This could be due to telomere‐independent functions of telomerase (Ale‐Agha et al., 2014; Chiodi & Mondello, 2012). For example, in conditions of oxidative stress, telomerase is translocated from the nucleus to the mitochondria, where it protects mitochondrial DNA and improves mitochondrial function (Ahmed et al., 2008; Martens et al., 2019). Similarly, in conditions of proteotoxic stress, telomerase prevents apoptosis (Zhou et al., 2014). Telomerase has also been shown to increase the rate of cell proliferation (Gonzalez‐Suarez et al., 2001; Hrdlickova et al., 2012; L. L. Smith et al., 2003), possibly via activation of c‐Myc and Wnt (Choi et al., 2008) or via inhibition of TGFβ (Stampfer et al., 2001). Telomerase‐driven increase in cell proliferation could not only counteract the features of cellular aging such as a reduction in proliferation rate and an increase in soma size, but could also reduce damage accumulation. It has been hypothesized that damage accumulation is modulated by proliferation: during mitosis, cells can dilute damage in a symmetric or an asymmetric manner (Hill et al., 2017; Ogrodnik et al., 2014). Is summary, the role of telomerase in counteracting cellular aging goes beyond telomere lengthening, but more research is needed to assess the contribution of telomerase functions on the kinetics of cellular aging.

## CONFLICT OF INTEREST

None declared.
